# Decay kinetics of HIV-1-RNA in seminal plasma with dolutegravir/lamivudine versus dolutegravir plus emtricitabine/tenofovir alafenamide in treatment-naive people living with HIV

**DOI:** 10.1093/jac/dkad245

**Published:** 2023-08-07

**Authors:** Abraham Saborido-Alconchel, Ana Serna-Gallego, Luis E Lopez-Cortes, María Trujillo-Rodriguez, Juan Manuel Praena-Fernandez, Montserrat Dominguez-Macias, Carmen Lozano, Esperanza Muñoz-Muela, Nuria Espinosa, Cristina Roca-Oporto, Cesar Sotomayor, Marta Herrero, Alicia Gutierrez-Valencia, Luis F Lopez-Cortes

**Affiliations:** Infectious Diseases and Microbiology Clinical Unit . Instituto de Biomedicina de Sevilla/University Hospital Virgen del Rocío/CSIC/Universidad de Sevilla. Sevilla. Spain; Infectious Diseases and Microbiology Clinical Unit . Instituto de Biomedicina de Sevilla/University Hospital Virgen del Rocío/CSIC/Universidad de Sevilla. Sevilla. Spain; Infectious Diseases and Microbiology Clinical Unit (Department of Medicine, School of Medicine). Instituto de Biomedicina de Sevilla/University Hospital Virgen. Seville. Spain. Macarena/CSIC/Universidad de Sevilla . CIBERINFEC, Instituto de Salud Carlos III, Madrid, Spain; Infectious Diseases and Microbiology Clinical Unit . Instituto de Biomedicina de Sevilla/University Hospital Virgen del Rocío/CSIC/Universidad de Sevilla. Sevilla. Spain; Departamento de Estadística e Investigación Operativa. Facultad de Ciencias . Universidad de Granada. Spain; Infectious Diseases and Microbiology Clinical Unit . Instituto de Biomedicina de Sevilla/University Hospital Virgen del Rocío/CSIC/Universidad de Sevilla. Sevilla. Spain; Infectious Diseases and Microbiology Clinical Unit . Instituto de Biomedicina de Sevilla/University Hospital Virgen del Rocío/CSIC/Universidad de Sevilla. Sevilla. Spain; Infectious Diseases and Microbiology Clinical Unit . Instituto de Biomedicina de Sevilla/University Hospital Virgen del Rocío/CSIC/Universidad de Sevilla. Sevilla. Spain; Infectious Diseases and Microbiology Clinical Unit . Instituto de Biomedicina de Sevilla/University Hospital Virgen del Rocío/CSIC/Universidad de Sevilla. Sevilla. Spain; Infectious Diseases and Microbiology Clinical Unit . Instituto de Biomedicina de Sevilla/University Hospital Virgen del Rocío/CSIC/Universidad de Sevilla. Sevilla. Spain; Infectious Diseases and Microbiology Clinical Unit . Instituto de Biomedicina de Sevilla/University Hospital Virgen del Rocío/CSIC/Universidad de Sevilla. Sevilla. Spain; Infectious Diseases and Microbiology Clinical Unit . Instituto de Biomedicina de Sevilla/University Hospital Virgen del Rocío/CSIC/Universidad de Sevilla. Sevilla. Spain; Infectious Diseases and Microbiology Clinical Unit . Instituto de Biomedicina de Sevilla/University Hospital Virgen del Rocío/CSIC/Universidad de Sevilla. Sevilla. Spain; Infectious Diseases and Microbiology Clinical Unit . Instituto de Biomedicina de Sevilla/University Hospital Virgen del Rocío/CSIC/Universidad de Sevilla. Sevilla. Spain

## Abstract

**Background:**

This was a substudy of a Phase IV, randomized clinical trial (ClinicalTrials.gov identifier: NCT04295460) aiming to compare the activity of dolutegravir/lamivudine versus dolutegravir plus tenofovir alafenamide/emtricitabine (DTG + TAF/FTC) in the male genital tract.

**Methods:**

Participants were asymptomatic adults without sexually transmitted diseases, treatment-naive people living with HIV (PLWH), with CD4+ T cell counts >200 cells/mm^3^ and plasma HIV-1-RNA levels >5000 and <500 000 copies/mL, randomized (1:1) to DTG + TAF/FTC or dolutegravir/lamivudine. Blood plasma (BP) and seminal plasma (SP) were collected at baseline and Weeks 4, 8, 12 and 24. HIV-1-RNA was measured in BP and SP using the Cobas 6800 system (Roche Diagnostics) with a lower detection limit of 20 copies/mL. The primary efficacy endpoint was the proportion of subjects with undetectable SP HIV-1-RNA at Week 12 by intention-to-treat analysis.

**Results:**

Fifteen participants in the DTG + TAF/FTC and 16 in the dolutegravir/lamivudine arms were analysed, with basal SP viral load of 4.81 (4.30–5.43) and 4.76 (4.09–5.23), *P* = 0.469, respectively. At Week 12, only one participant in each treatment arm had a detectable SP HIV-1-RNA (DTG + TAF/FTC, 141 copies/mL; dolutegravir/lamivudine, 61 copies/mL). Based on the estimated means, there was no significant difference in the decay of HIV-1-RNA in both BP and SP over time between the two arms of treatment (F = 0.452, *P* = 0.662, and F = 1.147, *P* = 0.185, respectively).

**Conclusions:**

After 12 weeks of treatment, there were no differences in the percentage of undetectable SP HIV-1-RNA in naive PLWH who started dolutegravir/lamivudine compared with DTG + TAF/FTC.

## Introduction

Higher plasma and genital HIV-1-RNA concentrations are associated with a higher risk of HIV-1 transmission to serodiscordant partners, with rare transmission rates with plasma HIV-1-RNA below 1500 copies/mL.^1,2^ However, it is unknown whether there is a certain threshold of viral load in seminal plasma (SP) below which transmission does not occur. In recent years, a large body of clinical evidence has established that viral load ‘undetectable = untransmittable’ is scientifically sound.^3–7^ However, a residual risk of HIV transmission persists during the first months on ART, even with undetectable plasma viral load.^8^

In recent years, dual therapy with dolutegravir/lamivudine has shown similar virological efficacy to triple therapy based on dolutegravir in treatment-naive people living with HIV (PLWH), with lower cost and, probably, less long-term toxicity.^9^ However, there are still some questions about the efficacy of dolutegravir plus lamivudine, including its activity in the genital tract, compared with triple therapy. Herein, we aimed to compare the decay kinetics of HIV-1-RNA in SP in naive participants who started dolutegravir plus tenofovir alafenamide/emtricitabine (DTG + TAF/FTC) versus dolutegravir plus lamivudine.

## Materials and methods

### Study design and participants

This was a substudy of the investigator-initiated Phase IV, randomized, open-label clinical trial (ClinicalTrials.gov identifier: NCT04295460; EMA, N^o^ EudraCT: 2019-000800-14) conducted in several hospitals in Andalucía, Spain. Participants were asymptomatic MSM, treatment-naive PLWH older than 18 years, with CD4+ T cell counts >200 cells/mm^3^ and plasma HIV-1-RNA levels between 5000 and 500 000 copies/mL. Exclusion criteria included HIV resistance to the study drugs, active sexually transmitted infections screened at baseline (syphilis, gonorrhoea, *Chlamydia trachomatis* and *Mycoplasma genitalium*) or presence of symptoms later, opportunistic infection, HBV or HCV coinfection, cirrhosis, previous or current malignancies, treatment with immunomodulatory agents, and use of drugs with potential interactions with prescribed drugs. Eligible patients were centrally randomized (1:1) to initiate dolutegravir (50 mg) plus tenofovir alafenamide/emtricitabine (25/200 mg) or a single tablet of dolutegravir/lamivudine (50/300 mg) once daily. The study was carried out according to the principles of the Declaration of Helsinki. It was approved by the Spanish Agency for Medicines and Healthcare Products and the Ethics Committee for Research on Medicinal Products of Seville (record# 02/2020). All participants provided signed informed consent.

### Follow-up, assessments and samples

Patients were evaluated at baseline and 4, 8, 12 and 24 weeks after the initiation of treatment, including adherence, adverse effects, haematology and biochemistry tests, CD4+ and CD8+ T cell counts in fresh blood (FC 500 flow cytometer, Beckman Coulter, Brea, CA, USA). Participants collected semen samples into a dry sterile container about 1 h before blood samples were taken. Semen samples were processed within 2 h after collection. SP was separated by centrifugation and stored at −80°C until tested. Blood plasma (BP) and SP HIV-1-RNA levels were measured by PCR (Cobas 6800 system; Roche Diagnostics) with a lower detection limit of 20 copies/mL.

### Efficacy outcomes

In a previous study, we observed that with elvitegravir/cobicistat or rilpivirine plus tenofovir disoproxil fumarate and emtricitabine all participants reached an undetectable SP viral load at Week 12.^10^ Thus, the primary endpoint of this substudy was the proportion of subjects with undetectable HIV-1-RNA in SP at Week 12 of treatment, defined as <20 copies/mL. A secondary endpoint was differences in the time-weighted mean change in SP of HIV-1-RNA according to the treatment arm.

### Statistical analysis

Given the proof-of-concept nature of this study, no formal sample size was calculated. The results were expressed as medians and IQRs for continuous variables and numbers and percentages for categorical variables. The χ^2^ test, Fisher’s exact test and the Mann–Whitney *U* test were used to compare the baseline demographic and clinical characteristics by treatment arms. BP and SP values were log_10_-transformed, with undetectable HIV-1-RNA samples set at 1.20 log_10_ copies/mL. Correlations were assessed using the Spearman correlation coefficient. The SP HIV-1-RNA trend throughout the study period was evaluated using a generalized linear model for repeated measures to test the impact of the different treatments. The overall effect of treatment on the outcome variable (SP HIV-1-RNA decay) was tested using the F-test and 95% CIs. Interaction terms (treatment arm × time) were included to compare the slopes of changes in the outcome variable. As the sphericity condition was not met (Mauchly’s sphericity test), the Huynh–Feldt correction was interpreted for the effects within subjects. We used non-parametric techniques to detect the effects within-treatment (Friedman test) and between-treatment (Mann–Whitney *U*-test) design. Pairwise comparisons were performed using the Wilcoxon test with Bonferroni correction. Statistical analyses were performed with SPSS version 26.0 (IBM Corp., USA) and *P* values <0.05 were considered significant.

## Results

### Study population

Between 22 June 2020 and 22 August 2022, 31 participants agreed to participate in the semen viral kinetics substudy. They were randomly assigned to DTG + TAF/FTC (*n* = 15) or dolutegravir/lamivudine (*n* = 16). All participants completed the study with 100% adherence to ART, based on monthly self-report and return of unused medication, without significant adverse events. At baseline, in the dolutegravir/lamivudine arm, two participants had undetectable SP HIV-1-RNA, and another one had 1 500 000 copies/mL in SP with a plasma viral load of 47 900 copies/mL, a CD4+ T cell count of 514 cells/mm^3^ and a CD4+/CD8+ ratio of 0.51 with a negative HIV test 5 years before. The baseline demographics and clinical characteristics were well balanced between the treatment arms (Table 1).

**Table 1.** Baseline demographic and clinical characteristics

Characteristic	DTG + TAF/FTC (*n* = 15)	DTG/3TC (*n* = 16)	*P* value
Age, years	28 (24–43)	27 (24–35)	0.861
Risk for HIV-1 infection, MSM	15	14	
Smoker, yes	3 (20.0)	4 (25.0)	0.739
Recreational drugs, yes	1 (6.7)	4 (25.0)	0.333
BMI, kg/m^2^	23.5 (22.5–25.2)	22.6 (19.9–25.9)	0.512
CD4+ T cell counts/mm^3^	394 (239–558)	454 (347–638)	0.295
CD4+/CD8+ ratio	0.57 (0.28–1.08)	0.50 (0.42–0.73)	0.843
BP HIV-1-RNA, log_10_ copies/mL	4.81 (4.30–5.43)	4.76 (4.09–5.23)	0.469
SP HIV-1-RNA, log_10_ copies/mL	3.39 (2.78–3.54)	3.80 (2.51–5.21)	0.276

Data expressed as median (IQR) or *n* (%). DTG/3TC, dolutegravir/lamivudine.

**Figure 1.**
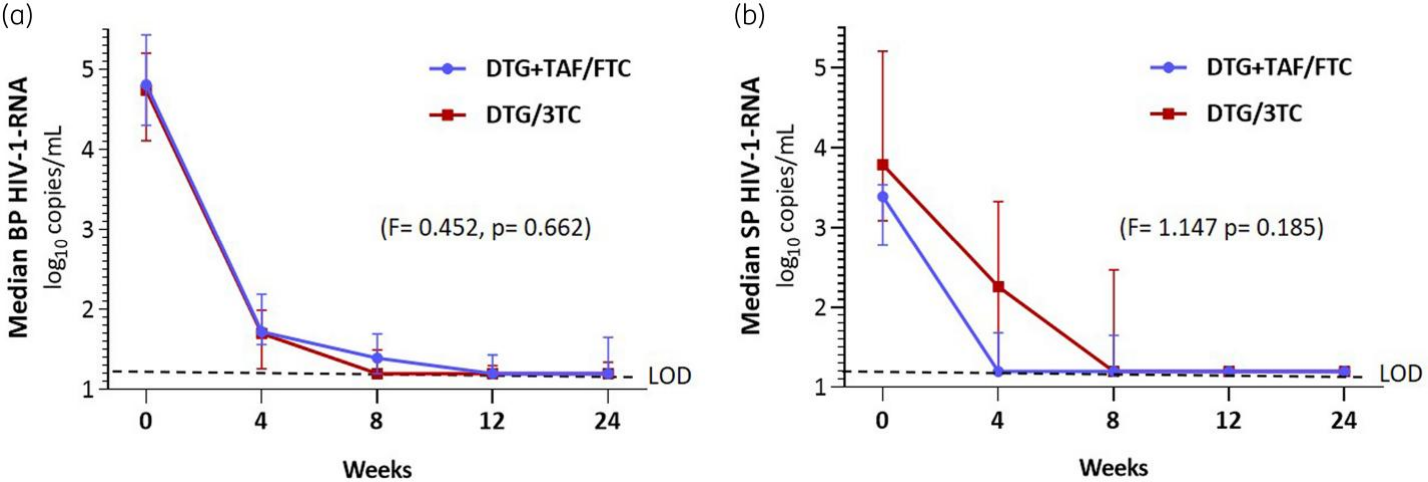
Median and IQR for (a) BP and (b) SP HIV-1-RNA log_10_ copies/mL as a function of the treatment received. Comparison of viral load decay after 12 weeks. DTG/3TC, dolutegravir/lamivudine. LOD, limit of detection (dotted line). This figure appears in colour in the online version of *JAC* and in black and white in the print version of *JAC*.

At baseline, there was a discrete correlation between basal HIV-1-RNA in BP and SP (ρ = 0.358, *P* = 0.048) that was no longer observed at subsequent timepoints due to a faster decay in BP than in SP. After 12 weeks, there were no differences in the decrease of BP HIV-1 RNA between the DTG + TAF/FTC and dolutegravir/lamivudine arms (F = 0.452, *P* = 0.662). Figure 1(a) shows the medians and IQRs in both treatment arms.

### Viral kinetics in semen

At Week 4, 11/15 participants on DTG + TAF/FTC showed undetectable SP HIV-1-RNA compared with 7/16 in the dolutegravir/lamivudine arm (*P* = 0.149). At Week 8, 11 out of 15 (73.3%) participants on DTG + TAF/FTC and 10 out of 16 (62.5%) on dolutegravir/lamivudine had undetectable SP HIV-1-RNA (*P* = 0.704). At Week 12, only one participant had detectable SP HIV-1-RNA in each treatment arm (DTG + TAF/FTC, 141 copies/mL; dolutegravir plus lamivudine, 61 copies/mL). At Week 24, in the DTG + TAF/FTC arm, one participant remained with a detectable SP viral load (93 copies/mL) and another showed a SP viral load rebound to 158 copies/mL with a previous negative determination. All participants in the dolutegravir/lamivudine arm showed undetectable SP HIV-1-RNA (*P* = 0.226) (Table 2). Figure 1(b) shows both treatment arms’ medians and IQRs of SP viral load. The generalized linear model results for repeated measures show no differences in the decay kinetics of the viral load in SP in both treatment arms after 12 (F = 1.747; *P* = 0.185) or 24 weeks. Table S1 (available as Supplementary data at *JAC* Online) shows the data without logarithmic transformation and the number of participants with HIV-1-RNA >200 copies/ml in SP.

**Table 2.** Decay kinetics of HIV-RNA in SP and cumulative incidence of participants with undetectable SP HIV-1-RNA according to the study design

	log_10_ copies/mL							
	Basal	Week 4	Dif. 0–4 week	Week 8	Dif. 0–8 week	Week 12	Dif. 0–12 week	Week 24
BP HIV-1-RNA								
* *DTG + TAF/FTC (*n* = 15)	4.81 (4.30–5.43)	1.72 (1.57–2.20)	3.19 (2.92–3.40)	1.40 (1.20–1.69)	3.45 (3.10–3.72)	1.20 (1.20–1.43)	3.55 (3.10–4.04)	1.20 (1.20–1.65)
	[3.86–5.66]	[1.20–2.32]	[2.08–3.68]	[1.20–1.95]	[2.86–4.07]	[1.20–2.25]	[2.66–4.46]	[1.20–1.81]
* *DTG/3TC (*n* = 16)	4.74 (4.12–5.20)	1.70 (1.26–2.00)	3.10 (2.88–3.44)	1.20 (1.20–1.49)	3.45 (2.92–3.85)	1.20 (1.20–1.30)	3.54 (3.10–4.04)	1.20 (1.20–1.34)
	[3.90–5.61]	[1.20–3.49]	[0.41–3.74]	[1.20–1.82]	[2.10–4.24]	[1.20–1.88]	[2.34–4.37]	[1.20–1.95]
* P* value	0.318	0.545	0.830	0.338	0.770	0.922	0.446	0.770
Undetectable BP HIV-1-RNA								
* *DTG + TAF/FTC, *n* (%)	0/15 (0.0)	11/15 (73.3)		11/15 (73.3)		14/15 (93.3)		13/15 (86.7)
* *DTG/3TC, *n* (%)	0/16 (0.0)	7/16 (43.8)		10/16 (62.5)		15/16 (93.8)		16/16 (100)
* P* value	1	0.192		0.794		0.4913		0.436
SP HIV-1-RNA								
* *DTG + TAF/FTC (*n* = 15)	3.39 (2.78–3.54)	1.20 (1.20–1.68)	1.71 (1.41–2.24)	1.20 (1.20–1.65)	2.14 (1.41–2.34)	1.20 (1.20–1.20)	2.19 (1.41–2.34)	1.20 (1.20–1.20)
	[2.41–5.59]	[1.20–3.51]	[0.84–2.518]	[1.20–2.51]	[1.13–3.07]	[1.20–1.81]	[0.97–4.39]	[1.00–2.20]
* *DTG/3TC (*n* = 16)	3.79 (3.09–5.21)	2.26 (1.20–3.33)	1.69 (0.66–2.34)	1.20 (1.20–2.47)	2.32 (1.39–3.18)	1.20 (1.20–1.20)	2.49 (1.68–4.01)	1.20 (1.20–1.20)
	[1.20–6.18]	[1.20–4.01]	[0.00–3.53]	[1.20–3.10]	[0.00–4.17]	[1.20–2.15]	[0.00–4.98]	[1.20–1.20]
* P* value	0.086	0.101	0.470	0.446	0.318	1	0.140	0.545
Undetectable SP HIV-1-RNA								
* *DTG + TAF/FTC, *n* (%)	0/15 (0.0)	11/15 (73.3)		11/15 (73.3)		14/15 (93.3)		13/15 (86.6)
* *DTG/3TC, *n* (%)	2/16 (12.5)	7/16 (53,8)		10/16 (62.5)		15/16 (93.8)		16/16 (100)
* P* value	0.484	0.149		0.704		1		0.226

Dif., difference; DTG/3TC, dolutegravir/lamivudine. Data expressed as median (IQR) and [range] unless otherwise stated. Undetectable HIV-1-RNA in BP was set to 1 log_10_ copies/mL. Undetectable HIV-1-RNA in SP was set to 1.20 log_10_ copies/mL.

## Discussion

This is the first controlled, randomized study to compare HIV-1-RNA decay in the SP of treatment-naive PLWH who started dolutegravir-based triple therapy versus dolutegravir plus lamivudine. As in other studies, BP HIV-1-RNA was higher than SP HIV-1-RNA, showing a discrete correlation between both compartments at baseline.^11,12^ Afterwards, such correlation was no longer observed due to a faster decay in BP than in SP.

Our results are consistent with those of clinical trials that have shown a similar viral load kinetics decay in BP with dolutegravir plus tenofovir disoproxil fumarate and emtricitabine as with dolutegravir/lamivudine.^9^ Here, we observed that both antiretroviral regimens had similar viral load kinetics decay in SP. At Week 12, only one sample was detectable in SP in each treatment arm, with values of 65 and 141 copies/mL, respectively. This low-level viral shedding in semen is probably below the threshold for sexual transmission.^13^

Regarding the probable causes for this similitude, the concentrations of lamivudine in the male genital tract might be sufficient to provide the same effect as tenofovir plus emtricitabine. Lamivudine accumulates in the male genital tract with a median SP/BP of 9.1 to 14.8 and median SP/BP AUC_0–12h_ ratio of 6.67, suggesting either active accumulation and/or inhibition of elimination.^14–16^ These concentrations are higher than those achieved by emtricitabine, whose AUC_0–24h_ ratio was 2.91,^17^ and even smaller concentrations of emtricitabine triphosphate in seminal cells than in PBMCs have been reported.^18^

On the other hand, tenofovir disoproxil fumarate has a very good penetration into the male genital tract, with a median SP/BP exposure ratio of 2.24 to 5.1 and intracellular concentrations of tenofovir diphosphate (the active metabolite) 17.5-fold higher than in BP.^19–21^ However, after tenofovir alafenamide administration, the tenofovir concentrations achieved in SP and the median tenofovir diphosphate seminal mononuclear cells/PBMCs ratio were about five times lower than those achieved with tenofovir disoproxil fumarate. Furthermore, the conversion of tenofovir alafenamide to tenofovir diphosphate appears to be slower, and its elimination is faster in semen than in blood.^22^ These facts could justify, at least in part, the similar results with both regimens.

Previously, seven studies with a single treatment arm have analysed the SP HIV-1-RNA decay from treatment-naive PLWH who started ART with an integrase strand transfer inhibitor (InSTI), five of them with dolutegravir, plus two nucleos(t)ide analogues, which results at Week 12 are shown in Table 3.^10,11,23–27^ Three of them showed similar results to those reported here.^10,23,24^ The studies by Ghosn *et al*.^11^ and by Mariaggi *et al*.^27^ differed from the others by including subjects with acute HIV infection and, therefore, with higher BP and SP viral loads.

**Table 3.** Studies analysing decay kinetics of HIV-1-RNA in SP with InSTI-based regimens

First author	*n*	Backbone	Third drug	Basal SP HIV-1-RNA log_10_ copies/mL	Undetectable SP HIV-1-RNA at Week 12 (%)
Imaz^15^	15	ABC/3TC	Dolutegravir	3.91 [2.97–4.82]	93.3
Gutierrez-Valencia^10^	12	TDF/FTC	Elvitegravir	3.88 [3.01–5.72]	100
Imaz^18^	15	TAF/FTC	Bictegravir	3.54 (2.41–3.79)	100
Fabrizio^16^	18	TDF/FTC or ABC/3TC	Dolutegravir	2.82 (1.89–3.13)	72.3
Fernandez-González^17^	12	ABC/3TC	Dolutegravir	4.11 (3.60–4.44)	58.3
Ghosn^12^	19	TDF/FTC	Dolutegravir	4.50 [2.70–5.90]	80.0
Mariaggi^13^	12	TDF/FTC	Dolutegravir	5.22 [3.45–6.21]	42.0
Current study	15	TAF/FTC	Dolutegravir	3.34 (2.70–3.56)	93.3
	14	3TC		3.80 (2.86–5.37)	92.9

Basal SP HIV-1-RNA expressed as median (IQR) median [range] log_10_ copies/mL.

The last-mentioned study showed the worst results, with 8/12 samples with detectable SP HIV-1-RNA at Week 12 but with a median of 35 copies/mL (IQR, undetectable to 93). In addition, Gianella *et al*. analysed 13 treatment- naive PLWH, but only results at Week 48 were reported, with only one participant showing 48 copies/mL.^28^ In all these studies in treatment-naive PLWH starting ART based on InSTI and the current one, all participants had SP HIV-1-RNA <200 copies/mL after 12 weeks of ART, suggesting that the time at risk for HIV sexual transmission by condomless sex after ART initiation with the new InSTIs could be shorter than previously estimated.^13,29^

Our study has some limitations. First, the sample size may be modest to obtain definitive conclusions; however, this is one with the largest sample size among the available studies. Second, the results are only applicable to men and not to women. However, it is expected that they are also relevant to women since the rate of HIV excretion in genital secretions and the viral load is usually lower than in men.^30–35^

In conclusion, there are no clinical differences in the decay kinetics of SP HIV-1-RNA in treatment-naive PLWH who started dolutegravir/lamivudine compared with DTG + TAF/FTC. After 12 weeks of treatment, all participants had an undetectable HIV-1-RNA or less than 200 copies/mL in SP. These results and the information in the available literature suggest that condomless sex may be safe after 12 weeks of treatment with the new InSTIs.

## Supplementary Material

dkad245_Supplementary_Data

## Transparency declarations

L.F.L.C. has received unrestricted research funding outside the submitted work from Gilead Sciences, Merck Sharp & Dohme, and ViiV Healthcare, and consultancy fees and lecture fees from Gilead Sciences and ViiV Healthcare. L.E.L.C. has served as a scientific advisor for Angelini, speaker for Angelini, ViiV Healthcare, Gilead Sciences and Correvio, and trainer for ViiV Healthcare. N.E. has received fees as a consultant and speaker from ViiV Healthcare, Merck Sharp & Dohme and Gilead Sciences, and travel grants from Gilead Sciences and Merck Sharp for attending conferences. M.T.R., A.S.G., M.D.M., E.M.M., C.S., M.H. and A.G.V. have received travel grants for attending conferences from Gilead Sciences.

## Supplementary data

Table S1 is available as Supplementary data at *JAC* Online.
