# Correction to: Decay kinetics of HIV-1-RNA in seminal plasma with dolutegravir/lamivudine versus dolutegravir plus emtricitabine/tenofovir alafenamide in treatment-naive people living with HIV

**DOI:** 10.1093/jac/dkad325

**Published:** 2023-11-03

This is a correction to: Abraham Saborido-Alconchel, Ana Serna-Gallego, Luis E Lopez-Cortes, María Trujillo-Rodriguez, Juan Manuel Praena-Fernandez, Montserrat Dominguez-Macias, Carmen Lozano, Esperanza Muñoz-Muela, Nuria Espinosa, Cristina Roca-Oporto, Cesar Sotomayor, Marta Herrero, Alicia Gutierrez-Valencia, Luis F Lopez-Cortes, Decay kinetics of HIV-1-RNA in seminal plasma with dolutegravir/lamivudine versus dolutegravir plus emtricitabine/tenofovir alafenamide in treatment-naive people living with HIV, *Journal of Antimicrobial Chemotherapy*, Volume 78, Issue 9, September 2023, Pages 2354–2360, https://doi.org/10.1093/jac/dkad245

In the originally published version of this manuscript, the authors’ affiliation information required correction.

Affiliations have now been updated online.

