# Supplementary material for: Decay kinetics of HIV-1-RNA in seminal plasma with dolutegravir/lamivudine versus dolutegravir plus emtricitabine/tenofovir alafenamide in treatment-naive people living with HIV
Source: J Antimicrob Chemother. 2023 Aug 7;78(9):2354–60. doi: 10.1093/jac/dkad245 (PMC10477137; doi:10.1093/jac/dkad245)
Supplement: dkad245_Supplementary_Data [file dkad245_supplementary_data.docx]

| **BP HIV-1-RNA**,  copies/mL | **Basal** | **Week 4** | **Week 8** | **Week 12** | **Week 24** |  |
| --- | --- | --- | --- | --- | --- | --- |
| **DTG+TAF/FTC** | 64800 (20000−273000) | 53 (37−156) | 25 (20−49) | 20 (20−27) | 20 (20-45) |  |
|  | [71800−462000] | [20−208] | [20−90] | [20−179] | [20−64] |  |
| **DTG/3TC** | 55800 (13225–160500) | 50 (22−100) | 20 (20−31) | 20 (20−25) | 20 (20−24) |  |
|  | [7960−412000] | [20−3118] | [20−66] | [20−75] | [20−90] |  |
| **p** | 0.451 | 0.780 | 0.591 | 0.780 | 0.652 |  |
| **SP HIV-1-RNA**,  copies/mL | **Basal** | **Week 4** | **Week 8** | **Week 12** | **Week 24** |  |
| **DTG+TAF/FTC** ^♣^ | 2480 (612−3540) | 20 (20−48) | 20 (20−45) | 20 (20−20) | 20 (20−20) |  |
|  | [260−388000] | [20−3270] | [20−327] | [20−65] | [20−158] |  |
| **DTG/3TC**^♦^ | 6249 (1417−190550) | 211 (20−2230) | 20 (20−298) | 20 (20−20) | 20 (20−20) |  |
|  | [20−1500000] | [20−2030] | [20−1250] | [20−141] | [20−20] |  |
| **p** | 0.009 | 0.046 | 0.320 | 0.983 | 0.983 |  |
| ♣ **>200 copies/ml** | 15/15 (100) | 3/15 (20.0) | 2/15 (13.3) | 0/15 (0.0) | 0/15 (0.0) | |
| ♦ **>200 copies/ml** | 14/16 (87.5) | 8/16 (50.0) | 5/16 (31.2) | 0/16 (0.0) | 0/14 (0.0) | |
| **p** | 0.493 | 0.171 | 0.445 | 1 | 1 |  |

**Supplementary Table 1**. Decay kinetics of HIV-RNA in seminal plasma (SP) and cumulative incidence of participants with undetectable SP HIV-1-RNA according to the study design. Undetectable HIV-1-RNA in BP and SP was set to 20 copies/ml. DTG+TAF/FTC, dolutegravir plus tenofovir alafenamide/emtricitabine. DTG/3TC: dolutegravir/lamivudine. Data expressed as median (IQR) and [range].
